# Outcomes of Sustained-Release Formulation of Valproate and Topiramate Monotherapy in Patients with Epilepsy: A Multi-Centre, Cohort Study

**DOI:** 10.1371/journal.pone.0047982

**Published:** 2012-12-11

**Authors:** Yida Hu, Xishun Huang, Dinglie Shen, Meiping Ding, Hongbin Sun, Bin Peng, Xiangshu Hu, Hua Li, Kebin Zeng, Zhiqin Xi, Ying Zhang, Qingqing Cao, Jing Liu, Yan Zhou, Mengjiao Wu, Yaodong Lu, Guojun Chen, Xuefeng Wang

**Affiliations:** 1 Department of Neurology, The First Affiliated Hospital, Chongqing Medical University, Chongqing, People's Republic of China; 2 Department of Neurology, The First Affiliated Hospital, Zhengzhou University, Zhengzhou, People's Republic of China; 3 Department of Neurology, Guangdong 999 Brain Hospital, Guangdong, People's Republic of China; 4 Department of Neurology, The Second Affiliated Hospital, Zhejiang University College of Medicine, Zhejiang, People's Republic of China; 5 Department of Neurology, Sichuan Academy of Medical Sciences and Sichuan Provincial People's Hospital, Sichuan, People's Republic of China; 6 School of Public Health, Chongqing Medical University, Chongqing, People's Republic of China; 7 Department of Cell Biology and Physiology, University of Pittsburgh School of Medicine, Pittsburgh, Pennsylvania, United States of America; University of British Columbia, Canada

## Abstract

**Background:**

New-generation antiepileptic drugs (AEDs) tend to replace traditional AEDs as the first-line choice for epilepsy. However, whether this change results in better outcome, especially in China, remains unknown.

**Methodology/Principal Findings:**

Two broad spectrum AEDs, the traditional drug of sustained-release formulation of valproate (SRVPA) and the new-generation drug of topiramate, were compared in patients with epilepsy as monotherapy in this multi-centre, observational cohort study from 2000 to 2011. The primary outcome was time to treatment failure. The secondary outcomes included time to first seizure, time to 12-month remission, and time to 24-month remission. Drug tolerability was assessed. Cox proportional hazard models (95% confidence interval [CI]) were used to analyse the relative risks expressed as hazard ratios (HR).

Of the 1008 recruited patients, 519 received SRVPA and 489 received topiramate. SRVPA was better than topiramate (28.3% vs. 41.5%; HR = 0.62, [95% CI 0.49–0.77]; p<0.0001) in primary outcome, and in time to first seizure (56.1% vs. 69.3%; HR = 0.73, [95% CI 0.62–0.86]; p = 0.0002). No significant difference was observed between two groups in time to 12-month remission (52.6% vs. 42.5%; HR = 1.01, [95% CI 0.84–1.23]; p = 0.88) and time to 24-month remission (34.7% vs. 25.2%; HR = 1.11, [95% CI 0.88–1.42]; p = 0.38). 36 patients (6.9%) in SRVPA group and 37 patients (7.6%) in topiramate group presented treatment failure associated with intolerable adverse events, there was no significant difference between the two groups (p = 0.70).

**Conclusions:**

The SRVPA is more suitable than topiramate for Chinese epileptic patients, and our results support the viewpoint that traditional AEDs should be the first-line choice for epilepsy rather than new-generation AEDs.

## Introduction

Epilepsy is one of the most common neurological disorders, affecting approximately 50 million people worldwide [1]. Medications are still the most important therapeutic choice for seizure control. In clinical practice, the time of appearance of valproate is a label that physicians called valproate and the AEDs entering the market before valproate as traditional AEDs, and the AEDs being licensed after valproate as new-generational ones. Over the past 20 years, a number of new-generation antiepileptic drugs (AEDs) have been registered around the world. Compared with the traditional AEDs, the new-generation AEDs have pharmacokinetic and tolerability advantages and demonstrate less potential risk for teratogenicity [2], [3]. It becomes a trend that new-generation AEDs will replace traditional AEDs as the first-line choice for epilepsy. However, studies have shown discrepancies with respect to the persistence of the new-generation AEDs [4]–[6]. For example, a recent double-blind, randomised trial evaluated the efficacy, drug safety, and neuropsychological effects of the two oldest AEDs (ethosuximide and valproate) and one of the newest generation AEDs (lamotrigine) on childhood absence epilepsy and demonstrated that the older drugs were more effective than the new drug [7]. In different guidelines, which AEDs, the new-generation or the traditional ones, should be recommended as the first-line choice has been unsettled [2], [8].

International League Against Epilepsy (ILEA) proposed that choice of optimal AED for epilepsy should be based on randomized controlled trials (RCTs). Although RCTs could provide less biased results [3], the inherent limitations include excessively strict criteria for inclusion and exclusion, fixed titration schedules and relatively short periods of follow-up, thus limiting their daily clinical applications. Alternatively, an observational study may provide more pragmatic information [9], [10]. Indeed, much of our clinical and public health knowledge was from observational investigations [11].

In recent years, about ten new-generation AEDs have been registered in China, and the prescriptions of new drugs are rapidly increasing. Since previous studies have not provided a clear answer as to which generations of AED should be the first choice [12], [13], clinicians in China have gradually been in a habit of starting with the new-generation AEDs rather than the traditional AEDs. However, whether these new AEDs give rise to a better outcome remains unclear. Therefore, large scale, multi-centre, cohort studies have been carried out to compare the persistence of the two most frequently used AEDs, the traditional drug of sustained-release formulation of valproate (SRVPA) and the new-generation drug of topiramate, with the aim to give clinicians useful information to answer the question: is the new AEDs truly better than the traditional ones for Chinese epileptic patients?

## Methods

### Patients

This study was undertaken in one chartered city and four provinces of China: Chongqing City, Guangdong Province, Sichuan Province, Zhejiang Province and Henan Province. Based on the data of the sixth national census of population from National Bureau of statistics of China, the sample area covered a total population of 362 017 960 people. The beginning date of this census was 1st November, 2010, and the reporting date was 29th April, 2011. Recruitment of this study occurred from August 2000 to March 2010. The last follow-up visit was between March and July 2011. This study was approved by the Ethics Committee of Chongqing Medical University. All patients or their guardians provided written informed consent.

Patients with a definite diagnosis of epilepsy, treated with SRVPA or topiramate as monotherapy, between 2 to 75 years old, were enrolled in this study. The exclusion criteria included the following: epileptic syndromes; only acute symptomatic or non-epileptic seizures; a history of psychiatric or mood disorders; clinically significant laboratory abnormalities, including abnormal liver function, abnormal haematological system function, abnormal kidney function, abnormal endocrine system function, or heart disease; and clinician or the patient feeling that the treatment was contraindicated.

### Procedures

The information recorded during the first visit included patient demographics, information about previous antiepileptic treatment, history of febrile seizures, birth traumas, epilepsy in first-degree family members, and neurological diseases (e.g., stroke, head injury, cortical development disorder, or intracerebral infection). A general physical examination and a neurological examination were performed. Laboratory examinations were carried out. Electrocardiogram (ECG) examinations were also performed, if necessary. Surface electroencephalography was performed on each patient to detect significant changes that might contribute to diagnosis. Computed tomography, magnetic resonance imaging and additional examinations, such as thyroid hormones, autoantibodies, and rheoencephalography, were carried out if clinically needed. Clinicians were asked to classify the types of epilepsy, epileptic syndromes, and types of seizures according to the criteria of the ILAE [14], [15].

For the patients with a definite diagnosis of epilepsy, physicians prescribed them the AED which they could afford. And only the patients treated by the SRVPA or topiramate were enrolled in this study. The guidelines for the initial drug dose and titration were provided as follows. In children and adolescents (ages 2–16), the initial dosage of SRVPA was 10–15 mg/kg/day, with weekly increments of 5–10 mg/kg/day, and the target dosage was 20–30 mg/kg/day; the initial dosage of topiramate was 0.5–1 mg/kg/day, with weekly increments of 0.5–1 mg/kg/day, and the target dosage was 5–9 mg/kg/day. In adults, the starting dosage of topiramate was 25 mg per night, with a weekly increment of 25 mg/day, and the target dosage was 100–250 mg/day; the initial dosage of SRVPA was 500 mg/day, with a weekly increment of 250 mg/day, and the target dosage was 1000–2000 mg/day. In general, medications were given with small initial doses, and the doses were slowly increased until the seizures were under control. Efficacy and adverse events were balanced in the adjustment of the AED dosages.

The patients were asked to return for subsequent reviews at the second week, the first month, the third month, the sixth month and at successive half-year intervals from the date of initial medication. If clinical attention was necessary, more visits were scheduled between the regularly scheduled appointments. To control recall bias, each patient treated at our centres was asked to keep a medical diary with information on seizure onset, combinations with other drugs, adverse events, and hospital admissions. To control the loss of follow-up bias in the case of patients who did not appear for regular visits, follow-up data were obtained through telephone interviews or with structured questionnaire letters by mail. A patient who was lost of contact for more than one year was defined as a follow-up loss.

The primary outcome of this study was time to treatment failure (in addition to other AEDs due to lack of efficacy; discontinuation of SRVPA or topiramate due to lack of efficacy [LE], intolerable adverse events [IAEs], lack of efficacy combined with intolerable adverse events [LE & IAEs], poor compliance, patients' financial hardship, or a plan of pregnancy). A patient with poor compliance was defined as having discontinued SRVPA or topiramate treatment by his or her own volition. Secondary clinical outcomes were the time to first seizure, the time from SRVAP or topiramate treatment to achieve 12-month remission of seizures (patients without any type of seizure for at least 12 months), the time to 24-month remission of seizures and the drug tolerability. The incidence of clinically important adverse events (AEs) and the incidence of IAEs directly leading to treatment failure were analysed to investigate the outcome of drug tolerability.

Sample size calculations were based on the primary outcome. It was assumed that the treatment failure for SRVPA and topiramate were 25% and 35% after one year, respectively [16], [17]. In this study, SRVPA was considered as an active comparator, there needed 415 patients for each group to achieve 90% power (β = 0.1) at a 0.05 significance level to detect an equivalence hazard ratio of 1.35, assuming a dropout rate of 20% for both groups during the whole study.

### Statistical analysis

**Figure 1:**
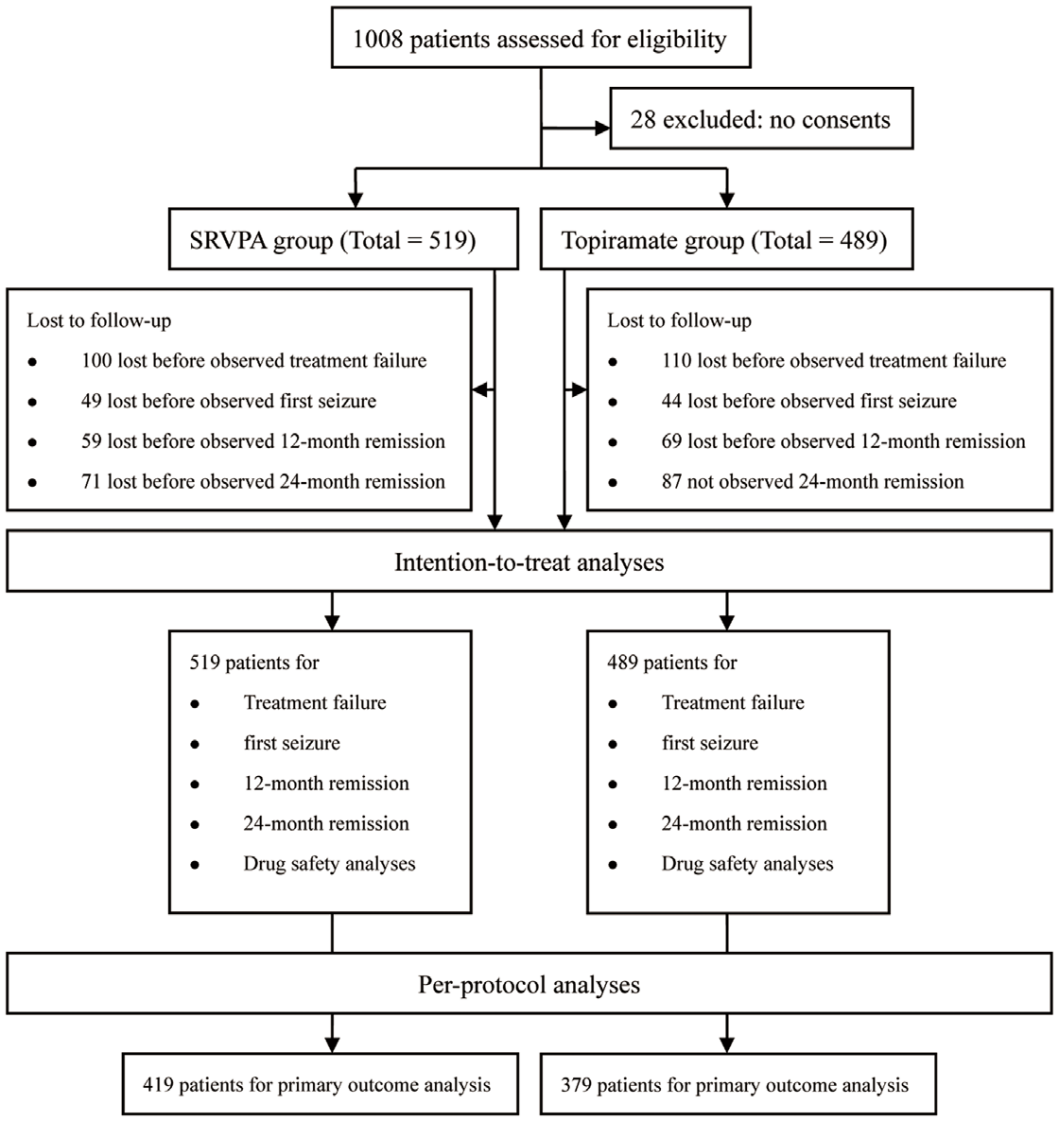
Study flow diagram.

The baseline characteristics of the two groups were compared using Chi-square tests and Fisher's exact tests, except the data represented as mean ± standard deviation (SD) which were analysed by using student's t-test. For the analysis of the primary and secondary outcomes, intention-to-treat (ITT) analysis was performed (Figure 1). The ITT population was defined as the population of all patients enrolled in this study. To roll out bias caused by the patients who were lost to follow-up, a per-protocol analysis was carried out to analyze the primary outcome. The PP population was defined as the population the in ITT analysis, excluding patients who were lost to follow-up before achieving the primary outcome. Kaplan-Meier estimates were used to describe the distribution of probability of non-treatment failure, probability of non-first seizure, probability of 12-month remission, and probability of 24-month remission. The log-rank tests were used to compare survival curves. The causes for censoring in Kaplan-Meier analysis were defined as follows: 1) for outcome of treatment failure: patients who were lost to follow-up, patients who died but whose death had no association with AED treatment and patients who were still receiving AED treatment at the end of this study; 2) for outcome of first seizure: patients who were lost to follow-up before their first seizures were observed, patients who died but whose death had no association with AED treatment, patients whose first seizures were not observed during this study, and patients whose first seizures had still not been observed by the time they suffered treatment failure; 3) for outcome of 12-month remission: patients who were lost to follow-up before they achieved 12-month remission, patients who died but whose death had no association with AED treatment, patients who did not succeed in achieving 12-month remission during this study, and patients who had still not achieved 12-month remission by the time they suffered treatment failure; 4) for outcome of 24-month remission: patients who were lost to follow-up before they achieved 24-month remission, patients who died but whose death had no association with AED treatment, patient who did not succeed in achieving 24-month remission during this study, and patients who had still not achieved 24-month remission by the time they suffered treatment failure. The censoring population was regarded as having no clinical outcome observed. Cox proportional hazard models (95% confidence interval) were used to analyse the relative risks expressed as hazard ratios (HR). In the final model, potential confounders (sex, age, type of epilepsy, seizure duration, number of previous AEDs and seizures at baseline) would be adjusted. Age was divided into three subgroups (2–16, >16 to 49, and >49 to 75). Seizure duration was defined as the difference between the age at the first seizure and the age at enrolment in this study and was divided into five subgroups (≤1 month, >1 month to 12 months, >12 months to 5 years, >5 years to 10 years, and >10 years). The number of previous AEDs was divided into four subgroups (no AEDs, one AED, two AEDs, and ≥three AEDs). Seizures at baseline denoted the number of seizures one month before the patients' participation in this study and were divided into four subgroups (no seizures, one seizure, two to three seizures, and ≥four seizures). Cox proportional hazard models that incorporated tests for interactions were used for all prespecified subgroup analyses. Tolerability was assessed in the ITT population. To compare the reasons leading to treatment failure between SRVPA and topiramate, Chi-square tests and Fisher's exact tests were used. All of the statistical analyses were performed with SPSS v. 13.0 software for Windows, using two-sided tests with a significance level of 0.05.

## Results

### Patient population

A total of 1008 patients were enrolled in this study (Figure 1). The study population had a mean age of 35.2±15.8 years for participation, with a majority being male (57.6%) with cryptogenic epilepsy (58.2%). A total of 210 patients were lost to follow-up before treatment failures were observed, of whom 100 (19.3%) were in the SRVPA group and 110 (22.5%) were in the topiramate group (p = 0.21). These patients were excluded from PP analysis for primary outcome. The patients in SRVPA group were younger than the patients in topiramate group (p<0.001). Also, their ages at first seizures were younger than the patients' in topiramate group (p<0.001). In contrast, the duration of follow-up of patients using SRVPA was statistically longer than that of patients using topiramate (p = 0.047). The baseline demographic and clinical characteristics of the patients are displayed in Table 1.

**Table 1:** Baseline demographic and clinical characteristics in patients treated with SRVPA^a^ or topiramate.

Characteristic	SRVPA(n = 519)	TPM^b^(n = 489)	p value
**Sex (n, %)**			0.21
Male	309 (59.5)	272 (55.6)	
Female	210 (40.5)	217 (44.4)	
**Age** c **(mean ± SD** d **, years)**	33.0±16.4	37.1±14.9	<0.001
**Age at seizure onset (mean ± SD, years)**	28.9±16·7	33.6±16.4	<0.001
**Duration of follow-up (median (range), months)**	24.0 (0.5–125.1)	18.0 (0.2–120.0)	0.047
**History of febrile convulsions (n, %)**	38 (7.3)	32 (6.5)	0.63
**Type of epilepsy (n, %)**			0.51
Idiopathic	30 (5.8)	36 (7.4)	
Symptomatic	180 (34.7)	175 (35.8)	
Cryptogenic	309 (59.5)	278 (56.9)	
**Type of Seizure (n, %)**			0.74
Simple partial	19 (3.7)	22 (4.5)	
Complex partial	40 (7.7)	29 (5.9)	
Secondary generalized	385 (74.2)	372 (76.1)	
Absence	5 (1.0)	3 (0.6)	
Tonic-Clonic	63 (12.1)	59 (12.1)	
Unclassified	7 (1.3)	4 (0.8)	
**Seizures at baseline** e **(n, %)**			0.79
No seizures	9 (1.7)	13 (2.7)	
1 seizure	311 (59.9)	289 (59.1)	
2–3 seizures	101 (19.5)	96 (19.6)	
≥4 seizures	98 (18.9)	91 (18.6)	
**Loss to follow-up before treatment failure**	100 (19.3)	110 (22.5)	0.21

a SRVPA = Sustained-release formulation of valproate.

b TPM = Topiramate.

c Defined as the age at the first visit of this study.

d SD = Standard deviation.

e Defined as the number of seizures one month before participating in this study.

### Primary outcome

**Figure 2:**
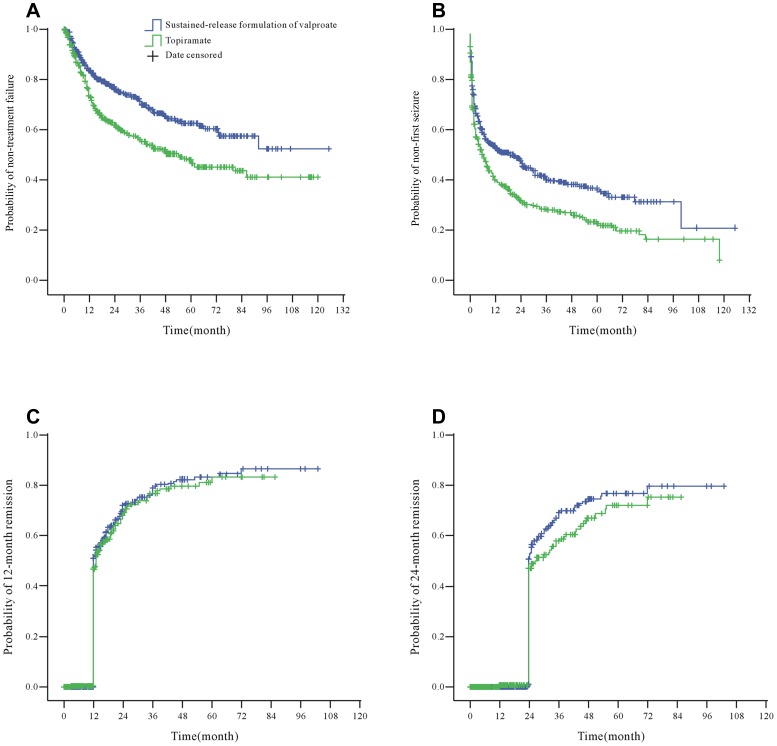
Kaplan-Meier estimates of clinical outcomes. A: Probability of non-treatment failure; B: Probability of non-first seizure; C: Probability of 12-month remission of seizures; D: Probability of 24-month remission of seizures.

In the ITT analysis, a total of 350 treatment failure events were observed over the whole study (Table 2). There was no statistical difference (p = 0.26) on median (25th–75th centile, months) of duration until failure between SRVPA group (11.0, 5.5–18.0) and topiramate group (11.0, 5.0–24.0). However, Kaplan-Meier analysis showed the patients treated with SRVPA had lower failure rates than those given topiramate (Figure 2.A). And, similar results were also confirmed by the Cox proportional hazard models (SRVPA vs. topiramate: 28.3% vs. 41.5%, after adjustment for potential confounders, HR = 0.62, 95% CI 0.49–0.77; p<0.001). The PP analysis results, both with (0.59, 0.48–0.74; p<0.001) and without adjustment (0.59, 0.48–0.73; p<0.001) were consistent with the results from ITT analysis..

**Table 2:** Intention-to-treat analysis of clinical outcomes according to SRVPA vs. topiramate.

Outcome	Number of patients (%)		Non-adjusted^a^		Adjusted^b^	
	SRVPA (n = 519)	TPM (n = 489)	HR^c^ (95% CI)	P value	HR (95% CI)	P value
Time to treatment failure	147 (28.3)	203 (41.5)	0.61 (0.50–0.76)	<0.001	0.62 (0.49–0.77)	<0.001
Time to first seizure	291 (56.1)	339 (69.3)	0.70(0.60–0.82)	<0.001	0.73 (0.62–0.86)	<0.001
Time to 12-month remission	273 (52.6)	208 (42.5)	1.05 (0.88–1.26)	0.57	1.01 (0.84–1.23)	0.88
Time to 24-month remission	180 (34.7)	123 (25.2)	1.18 (0.94–1.48)	0.17	1.11 (0.88–1.42)	0.38

a Did not adjust for potential confounders.

b Estimated from Cox proportional hazards models (95% confidence interval [CI]) after adjustment for sex, age, type of epilepsy, seizure duration, number of previous AEDs and seizures at baseline.

c HR = Hazard ratio.

The reasons for treatment failure in the SRVPA group and topiramate group are shown in Table 3. Of all treatment failure events in both groups, 236 (67.4%) were associated with inefficiency (both LE and LE & IAEs). Statistically, SRVPA was less likely to cause failures due to LE (16.8% vs. 28.2%) and financial hardship (0.2% vs. 1.6%) than topiramate. For treatment failures due to IAEs, LE & IAEs, and poor compliance, there were no significant differences between the two groups. 101 patients (19.5%) treated with SRVPA and 155 patients (31.7%) treated with topiramate complained about the inconvenience of taking their medication, and there was significant difference between them (p<0.001). During follow-up, three patients made plans for pregnancy, and all of them were in the SRVPA group. Table 4 showed that 50% patients in both groups seem to discontinue medication within 10 months due to LE and IAEs, and discontinue medication within 37 months due to poor compliance. There was no subgroup effect to modify the effect of the medication on the primary outcome. These results indicated the consistency of the AEDs' effects in different subgroups (table 5).

**Table 3:** Reasons for treatment failure in the SRVPA^a^ group and the topiramate group.

Reason	Number of patients (%)		RR^b^ (95% CI)	P value
	SRVPA (n = 519)	TPM (n = 489)		
Lack of efficacy	87 (16.8)	138 (28.2)	0.59 (0.47–0.75)	<0.001
Intolerable adverse events	32 (6.2)	30 (6.1)	1.05 (0.62–1.63)	0.98
Poor compliance^c^	20 (3.9)	20 (4.1)	0.94 (0.51–1.73)	0.85
LE & IAEs^d^	4 (0.8)	7 (1.4)	0.54 (0.16–1.83)	0.31
Financial hardship	1 (0.2)	8 (1.6)	0.12 (0.02–0.94)	0.02

a During follow-up, three patients made a plan for pregnancy, and all of them were in the SRVPA group.

b RR = Relative risk.

c Defined as the patient discontinuing SRVPA or topiramate treatment on his or her own volition.

d LE & IAEs = Lack of efficacy combined with intolerable adverse events.

**Table 4:** Median of duration until occurrence of outcomes.

	Median (25th–75th centile, months)		P value
	SRVPA	Topiramate	
**Outcomes**			
Treatment failure	11.0 (5.5–18.0)	11.0 (5.0–24.0)	0.26
First seizure	2.0 (1.0–7.0)	2.0 (0.5–8.0)	0.96
12-month remission	12.0 (12.0–13.6)	12.0 (12.0–13.0)	0.55
24-month remission	24.0 (24.0–24.2)	24.0 (24.0–24.0)	0.34
**Reasons for treatment failure**			
Lack of efficacy	9.0 (5.0–15.7)	10.0 (5.4–14.6)	0.94
Intolerable adverse events	7.0 (3.0–22.5)	9.9 (2.0–22.5)	0.91
Poor compliance^c^	36.7 (24.4–48.3)	31.3 (24.7–46.6)	0.91

**Table 5:** Subgroup analysis according to SRVPA vs. topiramate for the primary outcome.

Characteristic	No. of patients^a^		No. of events (%)^b^		HR (95% CI)	P value^e^
	SRVPA	TPM	SRVPA	TPM		
**Overall**	519	489	147 (28.3)	203 (41.5)	0.62 (0.49–0.77)	
**Sex**						0.49
Male	309	272	81 (26.2)	108 (39.7)	0.54 (0.40–0.73)	
Female	210	217	66 (31.4)	95 (43.8)	0.70 (0.50–0.98)	
**Age** c				0.58		
2–16	158	54	37 (23.4)	21 (38.9)	0.50 (0.28–0.92)	
>16 to 49	321	379	105 (32.7)	169 (44.6)	0.65 (0.51–0.83)	
>49 to 75	40	56	5 (12.5)	13 (23.2)	0.34 (0.10–1.12)	
**Type of epilepsy**						0.86
Idiopathic	30	36	8 (26.7)	13 (36.1)	0.52 (0.19–1.39)	
Symptomatic	180	175	50 (27.8)	63 (36.0)	0.52 (0.34–0.77)	
Cryptogenic	309	278	89 (28.8)	127 (45.7)	0.61 (0.46–0.81)	
**Seizure duration** d						0.15
≤1 month	44	41	10 (22.7)	14 (34.1)	0.48 (0.20–1.13)	
>1 month to 12 months	135	94	34 (25.2)	32 (34.0)	0.59 (0.35–1.00)	
>12 months to 5 years	162	141	36 (22.2)	55 (39.1)	0.59 (0.37–0.94)	
>5 years to 10 years	88	90	31 (35.2)	46 (51.1)	0.43 (0.26–0.69)	
>10 years	90	123	36 (40.0)	56 (45.5)	0.91 (0.59–1.40)	
**Number of previous AEDs**						0.25
None	299	237	66 (22.1)	93 (39.2)	0.46 (0.33–0.65)	
1 AED	130	127	43 (33.1)	52 (40.9)	0.74 (0.48–1.14)	
2 AEDs	55	83	20 (36.4)	31 (37.3)	0.88 (0.46–1.66)	
≥3 AEDs	35	42	18 (51.4)	27 (64.3)	0.62 (0.32–1.21)	
**Seizures at baseline**						0.53
No seizures	9	13	1 (11.1)	4 (30.8)	0.65 (0.06–6.51)	
1 seizure	311	289	83 (26.7)	103 (35.6)	0.71 (0.53–0.96)	
2–3 seizures	101	96	28 (27.7)	42 (43.8)	0.62 (0.37–1.04)	
≥4 seizures	98	91	35 (35.7)	54 (59.3)	0.47 (0.29–0.76)	

a No. of patients = Number of patients.

b No. of events = Number of treatment failure events in each subgroup.

c Defined as the age at the first visit of this study.

d Defined as the difference between the age at the patient's first seizure and the age at enrolment in this study.

e Defined as p value for interaction.

### Secondary outcome

The ITT analysis showed that the time to first seizure was significantly different between the SRVPA group and the topiramate group (log-rank statistic = 19.98, df = 1, p<0.001) (Figure 2.B). Table 2 showed fewer patients in the SRVPA group experienced a first seizure during the course of this study than in the topiramate group (56.1% vs. 69.3%). Overall, 481 patients (47.7%) achieved a 12-month remission, and 303 patients (30.1%) achieved a 24-month remission. No significant difference was observed between the SRVPA and topiramate groups for time to 12-month remission (log-rank statistic = 0.50, df = 1, p = 0.48) (Figure 2.C) or time to 24-month remission (log-rank statistic = 2.98, df = 1, p = 0.08) (Figure 2.D) by using Kaplan-Meier analyses. Similar results were confirmed by Cox analyses (Table 2).

### Safety and tolerability

Overall, 118 patients (22.7%) in the SRVPA group and 122 patients (24.9%) in the topiramate group reported clinically important AEs (Table 6). A total of 36 patients (6.9%) in the SRVPA group and 37 patients (7.6%) in the topiramate group demonstrated treatment failure due to IAEs or LE & IAEs, but there was no significant difference between the two groups (p = 0.70). The most common AEs associated with SRVPA treatment included dizziness, gastrointestinal reaction/appetite decrease (including nausea, vomiting or abdominal discomfort), extrapyramidal symptoms and somnolence. The most frequent AEs associated with topiramate included memory problems, limb anaesthesia, dizziness and somnolence. For SRVPA treatment, dizziness, weight gain and menstrual disorders were the most common IAEs leading to treatment failure. For topiramate, dizziness and memory problems were the most common IAEs associated with treatment failure.

**Table 6:** Clinically important adverse events and intolerable adverse events leading to treatment failure.

Adverse events	SRVPA (n = 519)		TPM (n = 489)	
	Clinically important^a^	Intolerable^b^	Clinically important	Intolerable
Total number (%) of patients^c^	118 (22.7)	36 (6.9)	122 (24.9)	37 (7.6)
Neurological/psychiatric system				
Dizziness	27 (5.2)	7 (1.3)	18 (3.7)	7 (1.4)
Extrapyramidal symptoms^d^	19 (3.7)	3 (0.6)	4 (0.8)	1 (0.2)
Somnolence	19 (3.7)	3 (0.6)	18 (3.7)	2 (0.4)
Memory problems	10 (1.9)	4 (0.8)	36 (7.4)	5 (1.0)
Headache	2 (0.4)	0 (0)	9 (1.8)	2 (0.4)
Tinnitus	2 (0.4)	0 (0)	0 (0)	0 (0)
Mental disturbance	1 (0.2)	0 (0)	7 (1.4)	3 (0.6)
Reaction dullness	1 (0.2)	0 (0)	6 (1.2)	1 (0.2)
Limb anaesthesia	0 (0)	0 (0)	20 (4.1)	3 (0.6)
Word-finding difficulty	0 (0)	0 (0)	12 (2.5)	1 (0.2)
Insomnia	1 (0.2)	0 (0)	1 (0.2)	1 (0.2)
Digestive system				
Gastrointestinal reaction/appetite decrease^e^	20 (3.9)	3 (0.6)	16 (3.3)	1 (0.2)
Abnormalities in liver function	3 (0.6)	1 (0.2)	1 (0.2)	1 (0.2)
Haematological system^f^	0 (0)	0 (0)	8 (1.6)	1 (0.2)
Others				
Weight gain	18 (3.5)	5 (1.0)	0 (0)	0 (0)
Weight decrease	0 (0)	0 (0)	13 (2.7)	2 (0.4)
Menstrual disorder	17 (3.3)	5 (1.0)	8 (1.6)	1 (0.2)
Alopecia	16 (3.1)	2 (0.4)	3 (0.6)	0 (0)
Fatigue	10 (1.9)	2 (0.4)	1 (0.2)	0 (0)
Skin rash	3 (0.6)	0 (0)	5 (1.0)	3 (0.6)
Haematuria	1 (0.2)	1 (0.2)	0 (0)	0 (0)
Calculus	0 (0)	0 (0)	6 (1.2)	3 (0.6)
Hypohidrosis	0 (0)	0 (0)	2 (0.4)	0 (0)

a Clinicians judged whether the adverse events recorded in the medical diaries of the patients were clinically important.

b IAEs = Intolerable adverse events leading to treatment failure. The patients who discontinued study AEDs due to lack of efficacy combined with intolerable adverse events were also included in this column.

c Denoted the total number of patients with at least one clinically important adverse event or intolerable adverse event.

d Extrapyramidal symptoms included tremor and abnormal gait.

e Gastrointestinal reactions included nausea, vomiting and abdominal discomfort.

f Haematological system abnormalities included thrombocytopoenia, leucopoenia, and anaemia.

## Discussion

The major finding of this study is that SRVPA was less likely to be associated with treatment failure than topiramate. No differences were found in terms of tolerability, time to 12-month remission, or time to 24-month remission between the two drugs. However, SRVPA was more suitable for Chinese epileptic patients to prevent first seizure than topiramate. These results do not support the viewpoint of starting with new-generation AEDs rather than traditional AEDs.

The change of prescription habits should be always based on the studies which could translate their findings into everyday use. However, few studies have answered which generation AEDs should be the first choice of initial monotherapy for Chinese epileptic patients. There was only one study have investigated the effectiveness of three AEDs for generalized onset and unclassified seizures in Chinese epileptic children retrospectively [18]. To design a study which could help clinicians judge whether starting with the new-generation drugs rather than traditional AEDs is reasonable, we consider the generalisability of the study results is the most important issue should be thought about carefully. To enhance the generalisability of the study results, firstly we chose SRVPA and topiramate as the drugs investigated, for they are the two most frequently used AEDs in China and they have the similar antiepileptic spectrum. Secondly, the database of this study included epileptic patients from one chartered city and four provinces of China, and the sample area covered a total population of 362 017 960 people. Thirdly, the range of participants was broad: the female and the male, from the children to the elderly, from the newly diagnosed to the refractory, from the patients on acute stage to the patients on the chronic stage were all included. In addition, the Han nationality is the largest among the 56 nationalities in China, occupying over ninety percent of the whole population. Accordingly, the results from our study are helpful to clinicians to establish medication strategy for the patients of Han nationality. The added value of this study may be important. Firstly, although treatment failure is considered as one of the best composite indicators for evaluating the long-term performance of AEDs, and this indicator had been recommended by the International League Against Epilepsy [19] in 1998, data regarding which drug is less likely to be associated with treatment failures on Chinese epileptic patients are relatively lacking. Secondly, the study design allowed clinicians to treat patients based on their routine practices, so that the results could be more applicable. Thirdly, as the epilepsy is a chronic disorder, short-term design may be difficult in assessing the real long-term persistence of AEDs. To the best of our knowledge, this is the longest study comparing the persistence of the SRVPA and the topiramate on Chinese epileptic patients.

For time to treatment failure, our study showed that the failure rates of topiramate were higher than those of valpriate, which was consistent with the results from the SANAD study (Arm B) [12]. However, a double-blind, randomised trial showed no difference in treatment failure between the two drug groups [13]. In the double-blind trial, the treatment dosage was fixed, and the period of follow-up was much shorter than that in our study, which could have led to the differences in outcome. Interestingly, our study showed that SRVPA was significantly less likely to cause discontinuation of treatment due to LE. This finding was in contrast to what was found by SANAD study, in which valproate and topiramate showed same treatment failure rate as a result of LE. In addition, our study showed that the LE, rather than AE is the most frequently precipitant for the discontinuations of SRVPA and topiramate, which is not consistent with other reports [12], [17], [20]. Compared with those studies, our study employed lower initial dosages and slower titration, and had longer time of follow-up. It has been demonstrated that a lower starting dosage and slower titration contribute to better drug tolerability [21]. It is notable that there was no difference on the median (25th–75th centile) of duration until failure between two groups. Both median of duration until failure were 11 months. From previous studies, we found the same phenomenon that no matter if there were failure rates differences or not, the median of duration until failure of AEDs were almost around 12 months [4], [11], [20]. For the main reasons leading to treatment failure in this study were lack of efficacy, intolerable adverse events, and poor compliance, we have calculated the reasons' median of duration until failure. We found that 50% patients in both groups seem to discontinue medication within 10 months due to lack of efficacy and intolerable adverse events, and discontinue medication within 37 months due to poor compliance. Since the number of patients suffered failure for inefficacy and adverse events was almost seven times as the number of patients discontinued treatment for poor compliance, that the median of duration until failure of both groups was 11 months was rational. Meanwhile, many studies have reported that IAEs often occurred early in treatment, whereas the timing of LE would take place much later [12], [20], [22]. And based on our results, although the median of duration until failure due to IAEs for both drugs was earlier than that due to LE, yet, the 75th centile of duration until failure caused by IAEs was much later than that caused by LE. This finding told us the occurrence of LE was in a relatively short period, while the IAEs could occur in a wide period during medication.

Seizure recurrence brings patients a great deal of mental burden. Also, time to seizure remission is one of the indicators to judge medication persistence. Thus, we added time to first seizure and time to 12- or 24-month seizure remission as the secondary outcomes of this study. Our results demonstrated that SRVPA was also better than topiramate in time to first seizure, although no differences were found in time to 12-month or 24-month remission. In addition, our study showed lower IAEs (both IAEs and LE & IAEs) rates than those in previous studies [4], [12], [13], [20]. In this study, 22·7% of patients in the SRVPA group and 24·9% of patients in the topiramate group reported adverse events. 6·9% and 7·6% of the patients in the two groups discontinued medications associated with IAE, respectively. The differences can be partly explained by the different prescription strategies and different population studied [22]. It is also notable that, topiramate rarely induces the haematological system abnormalities reported in previous studies [12], [20], [23], [24]. However, in our study, six patients presented with anaemia, one patient presented with dubious thrombocytopoenia, and one patient presented with granulocytopoenia. Nonetheless, most of the anaemia was very mild and transitory, and only one patient discontinued therapy. Currently, we have not been able to identify the direct relationship between topiramate use and haematological abnormalities.

The target dosages of SRVPA and topiramate were 1000–2000 mg/day and 100–250 mg/day, respectively. To achieve this dosage, the patients needed to take the drugs twice every day. Indeed, 19.5% of the patients treated with SRVPA and 31.7% of the patients treated with topiramate in our study complained about the inconvenience of taking their medication. Some patients stated that taking drugs twice a day meant doubling the risk of forgetting to take the drugs and doubling the worry that they would be known as epileptic patients by classmates or colleagues. Although there was no difference between the two medications in terms of treatment failure caused by poor compliance, this inconvenience should be noted by clinicians in practice.

### Limitations

Cohort studies have been used to examine a variety of outcomes after single exposure and are considered to be the best way to identify potential incidents [25], [26]. However, in an observational cohort study, unrandomised design may increase the risk of an imbalance of demographic characteristics between treatment groups and decrease the internal validity. To compensate for this difference, we used Cox proportional hazard models to adjust for potential confounders and tested the interaction effect between the subgroup indicators and the medications. Our results did not show evidence of interactions between the indicators and treatments. It is also notable that because the study was designed as unblinded, both clinicians and patients knew which treatment was chosen; this knowledge might have resulted in increased informative bias. To decrease the bias that came from the clinicians' preconceived ideas about the efficacy or adverse effects of the AED that was chosen, the clinicians were asked to collect information from laboratory examinations to detect possible adverse events. Meanwhile, patients were asked to keep regular medical diaries, which would help the clinicians to evaluate the primary and secondary outcomes more accurately.

Loss of follow-up was an important limitation for interpreting our results. During the long period of follow-up, 19·3% and 22·5% of patients were lost in SRVPA and topiramate groups, respectively. However, no difference was found between the two groups regarding the incidence of loss to follow-up, and both the PP analysis results and the ITT analysis results indicated that SRVPA is better than topiramate for primary outcomes.

### Conclusion

The effectiveness of the new-generational drug of topiramate is not superior to that of SRVPA. The SRVPA, one of the oldest broad-spectrum AEDs, by virtue of its lower long-term treatment failure rates and favourable efficacy profile in preventing occurrence of first seizures after medication, is still the optimal choice for Chinese patients with epilepsy. Lack of efficacy, rather than adverse events, was the most frequent reason for treatment failure, and the inconvenience of drug taking resulted in poor compliance, which was another important reason for treatment failure. In clinical practice, lower starting dosages and slower titration of AEDs will contribute to drug tolerability.

## Supporting Information

Materials S1 **The Protocol of this study.**
(DOC)

Materials S2 **The checklist of items for this cohort study based on STROBE statement.**
(DOC)
