# Supplementary material for: Outcomes of Sustained-Release Formulation of Valproate and Topiramate Monotherapy in Patients with Epilepsy: A Multi-Centre, Cohort Study
Source: PLoS One. 2012 Dec 11;7(12):e47982. doi: 10.1371/journal.pone.0047982 (PMC3519782; doi:10.1371/journal.pone.0047982)
Supplement: Materials S1 — The Protocol of this study. (DOC) [file pone.0047982.s001.doc]

**Protocol of Outcomes of sustained-release formulation of valproate and topiramate monotherapy in patients with epilepsy: a multi-centre, cohort study**

**Manuscript number: PONE-S-12-14140**

| **Study Title：** | Outcomes of sustained-release formulation of valproate and topiramate monotherapy in patients with epilepsy: a multi-centre, cohort study |
| --- | --- |
| **Clinical registration number：** | ChiCTR-OCH-12002147 |
| **Study Phase** | 4 |
| **Intervention** | Drug: sustained-release formulation of valproate (SRVPA)  Drug: topiramate (TPM) |
| **Indication** | Epilepsy |
| **Investigators** | The First Affiliated Hospital of Chongqing Medical University  The First Affiliated Hospital of Zhengzhou University  Guangdong 999 Brain Hospital  The Second Affiliated Hospital, Zhejiang University College of Medicine  Sichuan Academy of Medical Sciences & Sichuan Provincial People’s Hospital |
| **Sponsor** | The First Affiliated Hospital of Chongqing Medical University |
| **Study Director:** | Xuefeng Wang, MD |
| **Study Applicant** | Yida Hu, MD |

| Confidentiality Statement |
| --- |
| This confidential document is the property of The First Affiliated Hospital of Chongqing Medical University and it is provided for the use of the investigator and other designated personnel solely in connection with the conduct of the study described herein. No information contained herein may be disclosed, except as necessary to obtain consent from persons who are considering participation in the study, without prior written approval of The First Affiliated Hospital of Chongqing Medical University |

**TABLE OF CONTENTS**

[**1. BACKGROUND AND PURPOSE** 3](#__RefHeading___Toc325055557)

[**2. STUDY OUTCOMES** 4](#__RefHeading___Toc325055558)

[**2.1 Primary Outcome** 4](#__RefHeading___Toc325055559)

[**2.2 Secondary Outcomes** 4](#__RefHeading___Toc325055560)

[**3. METHODOLOGY** 5](#__RefHeading___Toc325055561)

[**3.1 Study Design** 5](#__RefHeading___Toc325055562)

[**3.2 Inclusion and Exclusion Criteria** 5](#__RefHeading___Toc325055563)

[**3.3 Schedule of Study Assessments for Subjects** 5](#__RefHeading___Toc325055564)

[**3.4 Medication** 8](#__RefHeading___Toc325055565)

[**3.5 Procedures** 8](#__RefHeading___Toc325055566)

[**3.6 Statistical Analysis** 9](#__RefHeading___Toc325055567)

[**3.6.1 Sample Size Calculation** 9](#__RefHeading___Toc325055568)

[**3.6.2 Analysis Populations** 9](#__RefHeading___Toc325055569)

[**3.6.3 Statistical Analysis Methods** 9](#__RefHeading___Toc325055570)

[**3.6.4 Subgroup analysis** 10](#__RefHeading___Toc325055571)

[**4 SAFTY** 10](#__RefHeading___Toc325055572)

[**4.1 Adverse Events, Severity, and Relationship** 10](#__RefHeading___Toc325055573)

[**4.2 Assessing Relationship to Study Treatment** 11](#__RefHeading___Toc325055574)

[**4.3 Classification of Causality** 12](#__RefHeading___Toc325055575)

[**4.4 Intolerable Adverse Events** 12](#__RefHeading___Toc325055576)

[**5 REMOVALS OF SUBJECTS FROM THE STUDY DRUG** 12](#__RefHeading___Toc325055577)

[**6. QUALITY CONTROL AND ASSURANCE** 13](#__RefHeading___Toc325055578)

[**7. ADMINISTRATIVE CONSIDERATIONS** 14](#__RefHeading___Toc325055579)

[**7.1 Ethical Conduct of the Study** 14](#__RefHeading___Toc325055580)

[**7.2 Subject Information and Consent** 14](#__RefHeading___Toc325055581)

[**7.3 Subject Confidentiality** 15](#__RefHeading___Toc325055582)

[**7.4 Protocol Violations/Deviations** 15](#__RefHeading___Toc325055583)

[**7.5 Access to Source Documentation** 15](#__RefHeading___Toc325055584)

**1. BACKGROUND AND PURPOSE**

With the registrations of new generation of antiepileptic drugs (AEDs) during the past 20 years, there seems to be a tendency that new generation AEDs will replace traditional AEDs as the first-line choice for epilepsy. However, studies have shown discrepancies with respect to their effectiveness. Even in different guidelines, which AEDs should be recommended as the first-line choice has been unsettled.

This cohort study aimed to evaluate the effectiveness of two most frequently used AEDs, the traditional sustained-release formulation of valproate (SRVPA) and the new-generation drug topiramate, to find evidence supporting the rationality of this tendency or evidence querying it.

**2. STUDY OUTCOMES**

**2.1 Primary Outcome**

The primary outcome of this study is time to treatment failure

Treatment failure is defined as: in addition to other AEDs due to lack of efficacy; discontinuation of SRVPA or topiramate due to lack of efficacy, intolerable adverse events, lack of efficacy combined with intolerable adverse events, poor compliance, patients’ financial hardship, or a plan of pregnancy. A patient with poor compliance was defined as having discontinued SRVPA or topiramate treatment by his or her own volition.

**2.2 Secondary Outcomes**

Secondary clinical outcomes are the time to first seizure, the time from SRVAP or topiramate treatment to achieve 12-month remission of seizures, the time to 24-month remission of seizures. The incidence of clinically important adverse events and the incidence of intolerable adverse events directly leading to treatment failure were also analysed

Patients achieve 12-month (or 24-month) remission of seizures are defined as patients without any type of seizure for at least 12 months (or 24 months).

**3. METHODOLOGY**

**3.1 Study Design**

Observational cohort design is to assess the effectiveness of SRVPA and TPM in Chinese epileptic patients.

For the patients with a definite diagnosis of epilepsy, an appropriate AED was chosen by the clinicians, taking into consideration the efficacy and adverse events of the available AEDs, the clinical characteristics and the economic status of the patient. The patients also had the right to refuse the clinicians’ prescriptions and could discuss the drugs with the clinicians to decide which AED would be chosen.

**3.2 Inclusion and Exclusion Criteria**

Inclusion criteria：Patients with a definite diagnosis of epilepsy, treated with SRVPA or TPM as monotherapy, between 2 to 75 years old, were enrolled in this study.

Exclusion criteria: epileptic syndromes; only acute symptomatic or non-epileptic seizures; a history of psychiatric or mood disorders; clinically significant laboratory abnormalities, including abnormal liver function, abnormal haematological system function, abnormal kidney function, abnormal endocrine system function, or heart disease; and clinician or the patient feeling that the treatment was contraindicated.

**3.3 Schedule of Study Assessments for Subjects**

| Study period | | Screen | Visit1 | Visit2 | Visit3 | Visit4 | Visit5 | Visit6 | Visit7 |
| --- | --- | --- | --- | --- | --- | --- | --- | --- | --- |
| Screen by inclusion/exclusion standard | | √ |  |  |  |  |  |  |  |
| Informed consent | | √ |  |  |  |  |  |  |  |
| Demographic information | | √ |  |  |  |  |  |  |  |
| Interrogation and physical examination | | √ | √ | √ | √ | √ | √ | √ | √ |
| Prescribing | |  | √ | √ | √ | √ | √ | √ | √ |
| EEG | | √ |  |  |  |  |  |  |  |
| Safety evaluation | Blood routine examination | IN* | IN | IN | IN | IN | IN | IN | IN |
|  | Urine routine examination | IN | IN | IN | IN | IN | IN | IN | IN |
|  | Liver function test | IN | IN | IN | IN | IN | IN | IN | IN |
|  | Kidney function test | IN | IN | IN | IN | IN | IN | IN | IN |
|  | ECG | IN | IN | IN | IN | IN | IN | IN | IN |
|  | Fasting blood sugar test | IN | IN | IN | IN | IN | IN | IN | IN |
|  | CT or MRI examination | IN | IN | IN | IN | IN | IN | IN | IN |
|  | Record adverse events described by patients |  | √ | √ | √ | √ | √ | √ | √ |
| Drop-out reason analysis | |  | √ | √ | √ | √ | √ | √ | √ |
| Drug combination judgment | | √ | √ | √ | √ | √ | √ | √ | √ |
| Evaluation | Efficacy |  |  | √ | √ | √ | √ | √ | √ |
|  | Compliance |  | √ | √ | √ | √ | √ | √ | √ |
|  | Safety |  | √ | √ | √ | √ | √ | √ | √ |

| Study period | | Visit8 | Visit9 | Visit10 | Visit11 | Visit12 | Visit13 |
| --- | --- | --- | --- | --- | --- | --- | --- |
| Screen by inclusion/exclusion standard | |  |  |  |  |  |  |
| Informed consent | |  |  |  |  |  |  |
| Demographic information | |  |  |  |  |  |  |
| Interrogation and physical examination | | √ | √ | √ | √ | √ | √ |
| Prescribing | | √ | √ | √ | √ | √ | √ |
| EEG | |  |  |  |  |  |  |
| Study period | | √ | √ | √ | √ | √ | √ |
| Safety evaluation | Blood routine examination | IN | IN | IN | IN | IN | IN |
|  | Urine routine examination | IN | IN | IN | IN | IN | IN |
|  | Liver function test | IN | IN | IN | IN | IN | IN |
|  | Kidney function test | IN | IN | IN | IN | IN | IN |
|  | ECG | IN | IN | IN | IN | IN | IN |
|  | Fasting blood sugar test | IN | IN | IN | IN | IN | IN |
|  | CT or MRI examination | IN | IN | IN | IN | IN | IN |
|  | Record adverse events described by patients | √ | √ | √ | √ | √ | √ |
| Drop-out reason analysis | |  | √ | √ | √ | √ | √ |
| Drug combination judgment | |  | √ | √ | √ | √ | √ |
| Evaluation | Efficacy | √ | √ | √ | √ | √ | √ |
|  | Compliance | √ | √ | √ | √ | √ | √ |
|  | Safety | √ | √ | √ | √ | √ | √ |

| Study period | | Visit14 | Visit15 | Visit16 | Visit17 | Visit18 | Visit19 |
| --- | --- | --- | --- | --- | --- | --- | --- |
| Screen by inclusion/exclusion standard | |  |  |  |  |  |  |
| Informed consent | |  |  |  |  |  |  |
| Demographic information | |  |  |  |  |  |  |
| Interrogation and physical examination | | √ | √ | √ | √ | √ | √ |
| Prescribing | | √ | √ | √ | √ | √ | √ |
| EEG | |  |  |  |  |  |  |
| Study period | | √ | √ | √ | √ | √ | √ |
| Safety evaluation | Blood routine examination | IN | IN | IN | IN | IN | IN |
|  | Urine routine examination | IN | IN | IN | IN | IN | IN |
|  | Liver function test | IN | IN | IN | IN | IN | IN |
|  | Kidney function test | IN | IN | IN | IN | IN | IN |
|  | ECG | IN | IN | IN | IN | IN | IN |
|  | Fasting blood sugar test | IN | IN | IN | IN | IN | IN |
|  | CT or MRI examination | IN | IN | IN | IN | IN | IN |
|  | Record adverse events described by patients | √ | √ | √ | √ | √ | √ |
| Drop-out reason analysis | |  | √ | √ | √ | √ | √ |
| Drug combination judgment | |  | √ | √ | √ | √ | √ |
| Evaluation | Efficacy | √ | √ | √ | √ | √ | √ |
|  | Compliance | √ | √ | √ | √ | √ | √ |
|  | Safety | √ | √ | √ | √ | √ | √ |

* IN = if necessary, which means: laboratory examinations were carried out. Electrocardiogram (ECG) examinations were also performed, if necessary.

If clinical attention was necessary, more visits were scheduled between the regularly scheduled appointments.

**3.4 Medication**

For this observational study, the medication program is totally in accord with the program which clinicians make in their routine practice. The guidelines for the initial drug dose and titration were provided as follows. In children and adolescents (ages 2-16), the initial dosage of SRVPA was 10-15 mg/kg/day, with weekly increments of 5-10 mg/kg/day, and the target dosage was 20-30 mg/kg/day; the initial dosage of topiramate was 0.5-1 mg/kg/day, with weekly increments of 0.5-1 mg/kg/day, and the target dosage was 5-9 mg/kg/day. In adults, the starting dosage of topiramate was 25 mg per night, with a weekly increment of 25 mg/day, and the target dosage was 100-250 mg/day; the initial dosage of SRVPA was 500 mg/day, with a weekly increment of 250 mg/day, and the target dosage was 1000-2000 mg/day. In general, medications were given with small initial doses, and the doses were slowly increased until the seizures were under control. Efficacy and adverse events were balanced in the adjustment of the AED dosages.

**3.5 Procedures**

The information recorded during the first visit included patient demographics, information about previous antiepileptic treatment, history of febrile seizures, birth traumas, epilepsy in first-degree family members, and neurological diseases (e.g., stroke, head injury, cortical development disorder, or intracerebral infection). A general physical examination and a neurological examination were performed. Laboratory examinations were carried out. Electrocardiogram (ECG) examinations were also performed, if necessary. Surface electroencephalography was performed on each patient to detect significant changes that might contribute to diagnosis. Computed tomography, magnetic resonance imaging and additional examinations, such as thyroid hormones, autoantibodies, and rheoencephalography, were carried out if clinically needed. Clinicians were asked to classify the types of epilepsy, epileptic syndromes, and types of seizures according to the criteria of the ILAE

The patients were asked to return for subsequent reviews at the second week, the first month, the third month, the sixth month and at successive half-year intervals from the date of initial medication. If clinical attention was necessary, more visits were scheduled between the regularly scheduled appointments. To control recall bias, each patient treated at our centres was asked to keep a medical diary with information on seizure onset, combinations with other drugs, adverse events, and hospital admissions. To control the loss of follow-up bias in the case of patients who did not appear for regular visits, follow-up data were obtained through telephone interviews or with structured questionnaire letters by mail. A patient who was lost of contact for more than one year was defined as a follow-up loss.

**3.6 Statistical Analysis**

**3.6.1 Sample Size Calculation**

Sample size calculations were based on the primary outcome. It was assumed that the treatment failure for SRVPA and topiramate were 25% and 35% after one year, respectively. In this study, SRVPA was considered as an active comparator, there needed 415 patients for each group to achieve 90% power (β=0.1) at a 0.05 significance level to detect an equivalence hazard ratio of 1.35, assuming a dropout rate of 20% for both groups during the whole study.

**3.6.2 Analysis Populations**

For the analysis of the primary and secondary outcomes, intention-to-treat (ITT) analysis was performed. The ITT population was defined as the population of all patients enrolled in this study. Because the ITT analysis included the data collected from patients who were lost to follow-up before treatment failure and because this fact might increase the likelihood of follow-up bias, a per-protocol (PP) analysis was carried out to detect the bias’ effect on the primary outcome. The PP population was defined as the population the in ITT analysis, excluding patients who were lost to follow-up before achieving the primary outcome.

**3.6.3 Statistical Analysis Methods**

Kaplan-Meier estimates were used to describe the distribution of time to treatment failure, time to first seizure, time to 12-month remission, and time to 24-month remission. The log-rank tests were used to compare survival curves. The causes for censoring in Kaplan-Meier analysis were defined as follows: patients who were lost to follow-up, patients who died but whose death had no association with AED treatment and patients who were still receiving AED treatment at the end of this study. The censoring population was regarded as having no clinical outcome observed. Cox proportional hazard models (95% confidence interval) were used to analyse the relative risks expressed as hazard ratios (HR). In the final model, potential confounders (sex, age, type of epilepsy, seizure duration, number of previous AEDs and seizures at baseline) would be adjusted. Tolerability was assessed in the ITT population. To compare the reasons leading to treatment failure between SRVPA and topiramate, Chi-square tests and Fisher’s exact tests were used. The baseline characteristics of the two groups were compared using nonparametric tests, except the data represented as mean ± standard deviation (SD), which were analysed by using student’s t-test. All of the statistical analyses were performed with SPSS v. 13.0 software for Windows, using two-sided tests with a significance level of 0.05. No interim analysis is planned for this study.

**3.6.4 Subgroup analysis**

Age was divided into three subgroups (2-16, >16 to 49, and >49 to 75). Seizure duration was defined as the difference between the age at the first seizure and the age at enrolment in this study and was divided into five subgroups (≤1 month, >1 month to 12 months, >12 months to 5 years, >5 years to 10 years, and ＞10 years). The number of previous AEDs was divided into four subgroups (no AEDs, one AED, two AEDs, and ≥ three AEDs). Seizures at baseline denoted the number of seizures one month before the patients’ participation in this study and were divided into four subgroups (no seizures, one seizure, two to three seizures, and ≥four seizures). Cox proportional hazard models that incorporated tests for interactions were used for all prespecified subgroup analyses.

**4 SAFTY**

Safety will be assessed by the nature, frequency, and severity of adverse events, vital signs, physical and neurological exams, blood and urine collections for clinical labs, and 12-Lead ECGs at the baseline (Visit 1) and over the scheduled visits during the study period. Changes from baseline will be displayed by central tendency, shift tables and marked abnormality. In addition, dropout rates, prior and concomitant medication usage and compliance rates will be calculated.

**4.1 Adverse Events, Severity, and Relationship**

Adverse Event (AE): Any untoward medical occurrence in a subject or clinical investigation subject administered an investigational product. An AE does not necessarily have a causal relationship with the medicinal product. For this study the medicinal product is SRVPA or TPM.

All AEs encountered during the clinical study will be reported on the record. All AEs, regardless of relationship to study drug or procedure, should be collected beginning from the time the subject signs the study consent. AEs in clinical investigation subjects include any change in the subject’s condition. This includes symptoms, physical findings, or clinical syndromes.

An abnormal laboratory value may be considered an AE if the identified laboratory abnormality leads to any type of intervention whether prescribed in the protocol or not. It is up to the investigator to determine whether an abnormal laboratory value constitutes an AE. If an abnormal laboratory value is caused by a disease process, the disease process and not the laboratory abnormality should be listed as the AE (eg, if new onset viral hepatitis is causing elevated ALT, hepatitis and not the elevated ALT should be listed as the AE).

Examples of laboratory abnormalities which should be considered as AEs include those which result in withdrawal of the study treatment, withholding study treatment pending some investigational outcome, reduction of dose of the study treatment, or additional concomitant treatment. All laboratory abnormalities considered to constitute an AE should be reported on the appropriate AE page of the record.

Laboratory abnormalities do not need to be listed as separate AEs if they are considered to be part of a clinical syndrome that is being reported as an AE. It is the responsibility of the investigator to review all laboratory findings in all subjects.

Abnormal values should be commented upon as to clinical relevance or importance on the record or the laboratory report as appropriate. Medical and scientific judgment should be exercised in deciding whether an isolated laboratory abnormality should be classified as an AE.

**Every effort must be made by the investigator to categorize each AE according to its severity and its relationship to the study treatment.**

**4.2 Assessing Relationship to Study Treatment**

Temporal relationship of the onset of the event to the initiation of the study treatment

The course of the event, considering especially the effect of discontinuation of study treatment or reintroduction of study treatment, as applicable

Whether the event is known to be associated with the study treatment, or with other similar treatments

The presence of risk factors in the study subject known to increase the occurrence of the event

The presence of non-study treatment related factors which are known to be associated with the occurrence of the event.

**4.3 Classification of Causality**

Not Related: A causal relationship between the study treatment and the adverse event is nota reasonable possibility.

Related: A causal relationship between the study treatment and the adverse event is a reasonable possibility.

**4.4 Intolerable Adverse Events**

Intolerable adverse event include any adverse event leading to treatment failure, such as: event results in death, event is life-threatening (Note: The term “life-threatening” refers to an event in which the subject was at risk of death at the time of the event; it does not refer to an event which hypothetically might have caused death if it were more severe.), event requires inpatient hospitalization or prolongation of existing hospitalization, event results in persistent or significant disability/incapacity, event is a congenital anomaly/birth defect and event leading to SRVPA or TPM withdraw.

**5 REMOVALS OF SUBJECTS FROM THE STUDY DRUG**

In accordance with the Declaration of Helsinki (48th General Assembly, Somerset West, Republic of South Africa, October 1996), subjects have the right to withdraw from the study at any time for any reason. Subjects may be removed from the study for the following reasons:

1. A serious or intolerable AE occurs

2. An intercurrent illness that, in the judgment of the investigator or sponsor, might invalidate the study or place the subject at risk.

3. At the request of the patients, whether economic or other reasons

If the reason for removal of a subject from the study is an AE, the AE and any related test results will be recorded on the record.

**When a subject is removed from the study:** the date of the last dose of study drug and all observations collected up to the time of termination will be recorded on the CRF along with the reason for termination

**6. QUALITY CONTROL AND ASSURANCE**

Before any subjects can be enrolled at a site, an initiation visit will be conducted for study personnel training. The investigator and the full relevant study site staff must be available at this visit. All staff must be trained on study procedures before they conduct any study specific procedures. Following the initiation visit, the study will be monitored approximately every 25 weeks (more when necessary) while subjects are actively enrolled into the study.

**7. ADMINISTRATIVE CONSIDERATIONS**

**7.1 Ethical Conduct of the Study**

The guidelines of the World Medical Association Declaration of Helsinki in its revised edition (48th General Assembly, Somerset West, Republic of South Africa, October 1996), the guidelines of ICH Good Clinical Practice (GCP) (CPMP/ICH/135/95), as well as the demands of national drug and data protection laws and other applicable regulatory requirements, will be strictly followed.

**7.2 Subject Information and Consent**

The investigator will inform the subject of the aims, methods, anticipated benefits, and potential hazards of the study, including any discomfort it may entail. The subject must be given every opportunity to clarify any points he/she does not understand and must be provided with more information if requested. At the end of the interview, the subject may be given time to reflect and can request more time if needed. The subject and/or legal guardian should consent participant into this study.

**7.3 Subject Confidentiality**

If applicable, the guidance set out in the European Directive 95/46/EC will be strictly adhered to when conducting clinical studies.

The subject must be assured that their identity will be protected. To facilitate this, a unique identification code will be assigned by the investigator to each study subject. This will be used instead of the subject’s name and cross-referenced with the subject’s date of birth when reporting AEs and /or other study-related data.

**7.4 Protocol Violations/Deviations**

In general, protocol violations include deviations from inclusion/exclusion criteria, from concomitant medication restrictions, and from any other protocol requirement that could, at least hypothetically, result in significant risk to the subject and/or affect the outcome of the study.

A deviation is defined as non-adherence to the study procedure or schedule as defined by the protocol, or the primary endpoint, that does not place the subject at any added or significant risk or affect the data quality or study outcome. Examples of protocol deviations that do not constitute protocol violations include a missed procedure, an out-of-window study visit, etc. Protocol violations will be noted in the final clinical report.

**7.5 Access to Source Documentation**

The investigator must permit the authorized sponsor, agents of the sponsor, and regulatory agency employees to enter and inspect any site where the drug or records pertaining to the drug are held, and to inspect and copy all records relating to an investigation including subject records.
